# Improving access to specialists in remote communities: a cross-sectional study and cost analysis of the use of eConsult in Nunavut

**DOI:** 10.1080/22423982.2017.1323493

**Published:** 2017-06-01

**Authors:** Clare Liddy, Fanny McKellips, Catherine Deri Armstrong, Amir Afkham, Leigh Fraser-Roberts, Erin Keely

**Affiliations:** ^a^ C.T. Lamont Primary Health Care Research Centre, Bruyère Research Institute, Ottawa, Ontario, Canada; ^b^ Department of Family Medicine, University of Ottawa, Ottawa, Ontario, Canada; ^c^ Department of Economics, University of Ottawa, Ottawa, Ontario, Canada; ^d^ Enabling Technologies, The Champlain Local Health Integration Network, Ottawa, Ontario, Canada; ^e^ Pediatrics, Children’s Hospital of Eastern Ontario, Ottawa, Ontario, Canada; ^f^ Department of Medicine, University of Ottawa, Ottawa, Ontario, Canada

**Keywords:** Primary care, eConsult, electronic consultation, wait times, rural communities, northern communities

## Abstract

**Background**: Residents of remote communities face inequities in access to specialists, excessive wait times, and poorly coordinated care. The Champlain BASE^TM^ (Building Access to Specialists through eConsultation) service facilitates asynchronous communication between primary care providers (PCP) and specialists. The service was extended to several PCPs in Nunavut in 2014.

**Objective**: To (1) describe the use of eConsult services in Nunavut, and (2) conduct a costing evaluation.

**Design**: A cross-sectional study and cost analysis of all eConsult cases submitted between August 2014 and April 2016.

**Results**: PCPs from Nunavut submitted 165 eConsult cases. The most popular specialties were dermatology (16%), cardiology (8%), endocrinology (7%), otolaryngology (7%), and obstetrics/gynaecology (7%). Specialists provided a response in a median of 0.9 days (IQR=0.3–3.0, range=0.01–15.02). In 35% of cases, PCPs were able to avoid the face-to-face specialist visits they had originally planned for their patients. Total savings associated with eConsult in Nunavut are estimated at $180,552.73 or $1,100.93 per eConsult.

**Conclusions**: The eConsult service provided patients in Nunavut’s remote communities with prompt access to specialist advice. The service’s chief advantage in Canada’s northern communities is its ability to offer electronic access to a breadth of specialties far greater than could be supported locally. Our findings suggest that a territory-wide adoption of eConsult would generate enormous savings.

## Introduction

Canada’s northern communities face numerous geographic, economic, and cultural barriers to accessing specialist care. Residents of these remote communities face inequities in access to service, excessive wait times, and poorly coordinated care. In Nunavut – a northern Canadian territory – the population is small and widely distributed. Many communities rely on nurses and nurse practitioners to provide the bulk of primary care services, supplemented by occasional visits from family physicians or a small group of core specialists (e.g. paediatricians), which may occur only a few times a year [1]. Beyond these infrequent visits, access to specialists often requires travel outside of the territory to cities thousands of kilometres away. These trips are rarely direct and may take a full day of travel, with frequent delays or cancellations due to poor weather conditions. This is especially true of trips from Iqaluit to larger cities in neighbouring provinces such as Ottawa, Ontario, which account for the bulk of time and costs [1]. In 2014/2015, the Government of Nunavut Department of Health reported spending more than a third of its total operational budget on medical travel [2]. In an effort to bridge these gaps in care, several telemedicine programmes have been implemented in northern Canada [3–5]. However, access remains a major issue in these regions, and poor internet connectivity can make the use of even the most efficient synchronous communication systems challenging [6].

The Champlain BASE^TM^ (Building Access to Specialists through eConsultation) eConsult service is a secure online platform that facilitates asynchronous communication between primary care providers (PCP) and specialists. Created in Ottawa, Canada, the eConsult service provides PCPs with prompt access to advice from 95 specialty groups. Responses arrive in a median of 0.8 days, and two-thirds of cases are resolved without patients requiring a face-to-face specialist visit [7].

Several Ottawa-based specialists enrolled with eConsult also provide clinical service to Nunavut, and advocated that the eConsult service be extended to the region. PCPs in Nunavut gained access to the eConsult service in August 2014.

In this study, we analysed all eConsult cases submitted by Nunavut PCPs in order to (1) describe the utilisation of the service for eConsults from Nunavut, and (2) conduct a costing evaluation to estimate the societal returns of the service. This is the first Canadian study on the use of eConsult in a northern region. Our results will be of interest to other health regions considering implementing an eConsult service.

## Methods

### Study design

This is a cross-sectional study and cost analysis of all eConsult cases submitted by participating PCPs located in Nunavut between August 2014 and April 2016.

### Setting

Nunavut is a territory in northern Canada with a population of 31,695 [8]. Communities range in size from several hundred to a few thousand people, with a demographic younger than the Canadian average. Each remote community has a health centre run by nurses, with physician consultation available by phone.

### The Champlain BASE^TM^ eConsult service

The eConsult service began as a small proof of concept in 2009–2010 and has grown into a full service funded by the Ontario Ministry of Health and Long-Term Care and research funding. To use the service, PCPs (which can include nurse practitioners as well as family physicians) log onto the web-based platform and enter their question along with pertinent clinical information into the appropriate fields. Relevant attachments can be included such as test results and images. PCPs can use the service in any Nunavut community, regardless of size. A designated case assigner allocates the case to a specialist in the desired specialty group based on availability. The specialist can request additional information or clarification, provide recommendations, or suggest a face-to-face consultation. Specialists are asked to reply within 7 days of receiving the request and are remunerated at a rate of $200 per hour prorated to their self-reported time required to complete the eConsult. The majority of participating specialists are located in the Champlain Local Health Integration Network (LHIN), a health region in Eastern Ontario, Canada.

### Data collection

**Figure 1.**
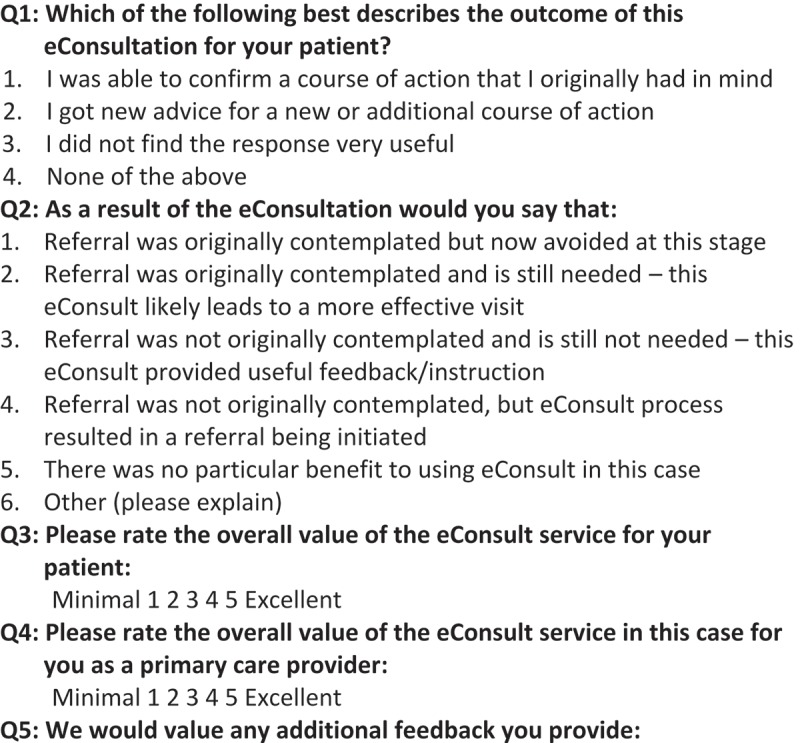
Mandatory closeout survey completed by primary care providers at the end of each eConsult.

The study drew on utilisation data collected automatically by the service, including demographic information on the specialist, PCP, and patient (e.g. date of birth, gender); the specialty group referred to; the dates of case submission, length of time to receive an initial response from specialists, and case closure; and specialists’ self-reported time spent answering the case. Data on case outcomes were obtained from a mandatory closeout survey completed by PCPs, which consists of four multi-choice questions and one optional free-text field, and captures information regarding eConsult’s impact on PCPs’ course of action, whether or not a face-to-face referral was avoided, and PCPs’ opinions regarding the value of the eConsult for the patient and themselves (Figure 1). Ethics approval for this study was provided by the Ottawa Health Science Network Research Ethics Board.

### Analysis

We collected descriptive statistics on all eConsults submitted by PCPs in Nunavut to describe their use of the eConsult service. For the costing evaluation, we estimated total societal costs and savings associated with eConsult. One eConsult was directed to a non-physician specialty (clinical pharmacist) and was excluded from the cost analysis. All costs and savings are reported in Canadian dollars and were adjusted to 2016 dollars when necessary using the Consumer Price Index [9].

We followed the same methodology to estimate the societal cost of eConsult as we used to estimate the cost implications of the original eConsult programme (2014–2015) in the Champlain region [10].

The total societal costs of eConsult included direct and indirect costs. Direct costs were those incurred by the payer while indirect costs are those incurred by the patient. Direct costs included (1) delivery costs, (2) consultation-specific costs, (3) added referral costs, and (4) patient travel costs. Delivery costs were those associated with supporting and operating the eConsult service and included administration costs, user support costs, and new user registration and training costs. Since fixed costs pertaining to administration (e.g. costs associated with billing) would have been incurred regardless of whether the PCPs were located in Nunavut or elsewhere, they were excluded from our total costs. On a per-case basis, these costs were negligible. We also excluded startup costs associated with eConsult, as our expansion to Nunavut allows us to leverage our existing service. These costs have been reported elsewhere [11].

Consultation-specific costs included specialist remuneration for answering eConsults as well as staff time for assigning cases to specific specialists. Staff time costs were based on a rate of $46 per hour. Added referral costs were calculated for eConsults where the PCP was not originally contemplating a referral, but initiated one as a result of eConsult (option 4 in question 2 of Figure 1). This cost was estimated using the general listing consultation fee for each specialty group set by the Ontario Ministry of Health and Long-Term Care and the visit was assumed to take place in Ottawa, Ontario as this would be the usual referral location for those cases. For these added referrals, patient travel costs were estimated based on the cost of a flight from the PCP’s town to Ottawa and back, using flight cost data from Canadian North airline [12]. We assumed that all patients under the age of 18 required adult accompaniment and added the cost of two flights. Our estimates were conservative, as accompaniment would likely be needed in other situations as well (e.g. patients with language barriers or physical disabilities). Indirect costs included patients’ lost wages for added referrals. We assumed patients travelling to Ottawa for a referral would miss 2 days of work – a conservative estimate considering that travel from many Nunavut communities takes more than 1 day per direction. We estimated the total amount of lost wages using the median wage in Nunavut [13].

The total potential societal savings due to eConsults were based on direct and indirect savings. Direct savings included (1) avoided referrals and (2) avoided travel costs. Avoided referrals were calculated for those where a face-to-face referral was avoided due to eConsult (option 1 in question 2 of Figure 1). For these avoided referrals, avoided patient travel costs were also calculated. Indirect savings consisted of patient wages that would have been lost had the referral not been avoided. All savings were estimated in the same way as the associated costs described above.

The overall potential societal cost savings were estimated by subtracting the total costs from the total savings.

In our main analysis, we considered added referrals as a cost. This was done for consistency. However, these added referrals constitute the avoidance of delay in a medical referral, which could have resulted in even greater costs to the patient and the payer due to poorer health outcomes and increased health system utilisation. Because these added referrals were not wasteful expenditure, we also reported the estimated cost savings of eConsult excluding these costs.

## Results

In total, 165 patients from Nunavut received an eConsult between August 2014 and April 2016. Most patients were adults between the ages of 18 and 65 (78%), while 13% were over 65 and 10% were under 18. The majority of cases (84%) came from Iqaluit, while the remainder came from Vernon (15%) and Clyde River (1%).

Nineteen PCPs registered to use the service, of whom 15 submitted at least one eConsult. While nurse practitioners are eligible to use eConsult, all eConsults submitted from Nunavut PCPs during the study period came from family physicians. Thirteen PCPs practised in Iqaluit, with the remaining two practising in Vernon and Clyde River, respectively.

**Figure 2.**
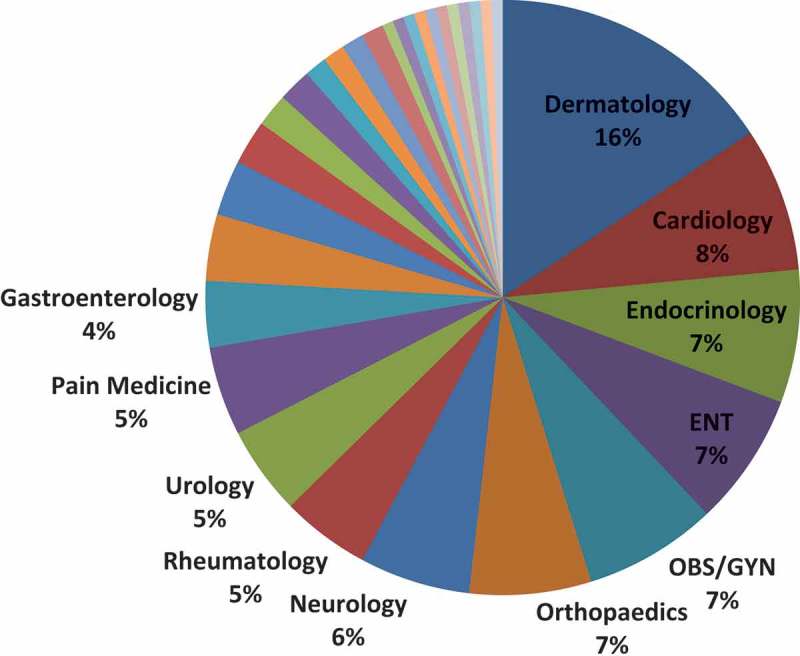
Specialty Distribution (N = 165) [OBS/GYN = obstetrics/gynaecology; ENT = otolaryngology].

PCPs submitted requests to 31 specialty groups and answered by 55 different specialists. The most commonly referred to specialties were dermatology (16%), cardiology (8%), endocrinology (7%), otolaryngology (7%), and obstetrics/gynaecology (7%) (Figure 2).

Specialists provided a response in a median of 0.9 days (IQR=0.3–3.0, range=0.01–15.02). The self-reported time specialists spent completing the eConsult was under 10 minutes in 38% of cases, 10–15 minutes in 23%, 15–20 minutes in 26%, and more than 20 minutes in 13%. Table 1 shows the average specialist self-reported billing time for eConsult cases by specialty.

**Table 1.** Average specialist self-reported time to complete eConsult and average specialist remuneration cost per eConsult (10 or more completed cases).

Specialty	N	Average Time to Complete (minutes)	Average Specialist Remuneration Cost per eConsult ($)
Orthopaedics	11	19.55	65.15
Dermatology	26	19.42	64.74
Endocrinology	12	14.58	48.61
AVERAGE		14.57	48.55
Neurology	10	13.50	45.00
Obstetrics/gynaecology	12	13.33	44.44
Cardiology	13	11.15	37.18
ENT	12	10.42	34.72

**Figure 3.**
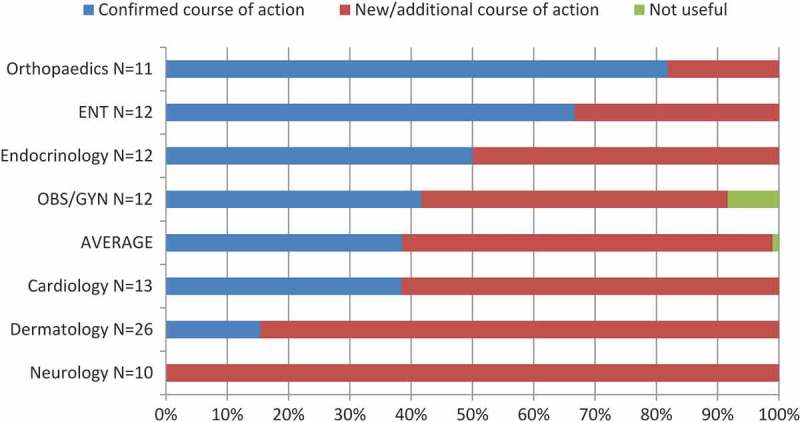
Impact of eConsult on course of action by specialty (10 or more completed cases) (n=96) [OBS/GYN=obstetrics/gynaecology; ENT=otolaryngology].

Overall, PCPs reported receiving new advice for an additional course of action in 56% of cases, while in 43% of cases the PCP was able to confirm the course of action they already had in mind. In 1% of cases, PCPs reported that the response was not very useful. The impact of eConsult on the course of action is shown by specialty in Figure 3.

**Figure 4.**
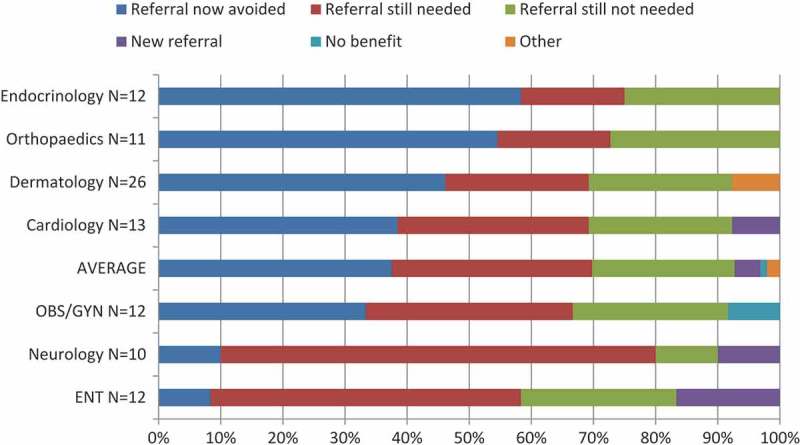
Impact of eConsult on need for face-to-face referral by specialty (10 or more completed cases) (n=96) [OBS/GYN=obstetrics/gynaecology; ENT=otolaryngology].

In 35% of cases, PCPs were able to avoid referring patients for a face-to-face specialist visit that they had originally planned. In particular, of the specialties which received more than 10 cases, endocrinology had the highest rate of avoided referrals (58%). Figure 4 shows the impact of eConsult on the need for a face-to-face referral by specialty.

PCPs rated the value of the eConsult service for themselves as 4 or 5 on a scale of 1 (minimal) to 5 (excellent) in 93% of cases, with an average score of 4.7/5. Using the same scale, they rated the value for patients as 4 or 5 in 93% of cases, with an average score of 4.6/5.

Optional feedback comments from PCPs are collected by the system during the closeout survey. These comments show that PCPs in Nunavut greatly appreciate the eConsult service. For example, one PCP wrote “excellent, timely feedback with additional teaching which will help me going forward in my career.” Another said “SUPER useful consult for a client with significant difficulty due to mobility issues and remoteness. Thank you!”

### Cost analysis

The delivery costs of eConsult in Nunavut were $1,379.69, including user setup and registration, user support, and variable administration costs. The cost of remunerating specialists was $8,600.00 and the total assignment costs were $31.43. Table 1 reports the average specialist remuneration cost per specialty. The physician/specialist fees associated with added referrals amounted to $539.15. Patient travel costs for these referrals were estimated at $13,088.25, while patient lost wages were estimated at $1,193.58. The total estimated costs of eConsult came to $24,832.10.

**Figure 5.**
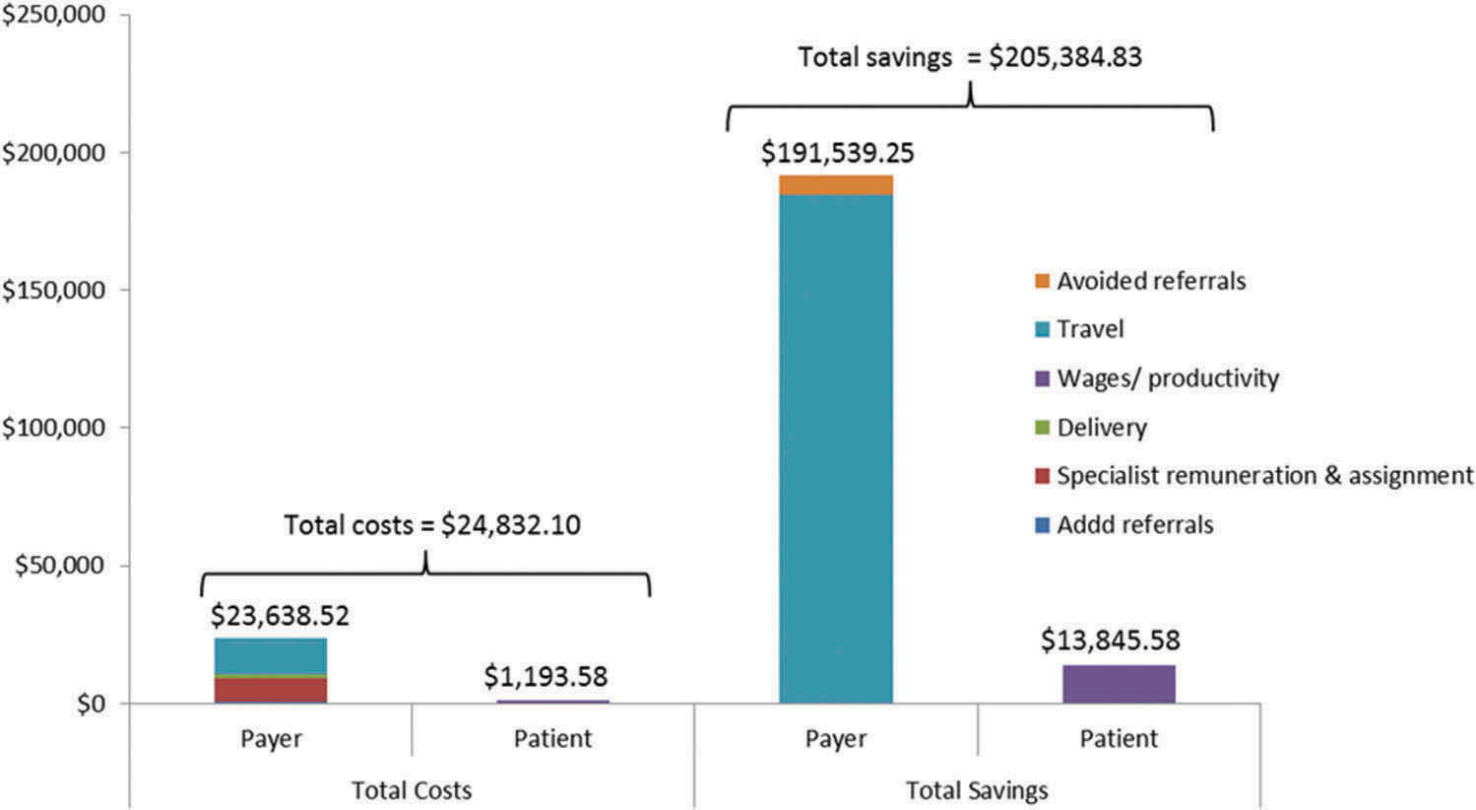
Stacked bar chart illustrating total societal costs (left) and total societal savings (right) for the Champlain BASE eConsult service.

Fifty-eight specialist referrals were no longer needed as a result of eConsult, which led to a saving of $7,092.05 in specialist fees. Avoided patient travel for these cases amounted to $184,447.20, while avoided patient lost wages came to $13,845.58. The total estimated savings resulting from eConsult were $205,384.83 (Figure 5).

The estimated total societal savings resulting from eConsult in Nunavut were $180,552.73, or $1,100.93 per eConsult. Excluding the costs of added referrals from the cost analysis increased the estimated societal savings to $195,373.71 or $1,191.30 per eConsult.

## Discussion

The eConsult service provided prompt access to specialist care for patients in Nunavut’s remote communities. Patients received access to specialist advice within days, and a third of cases resulted in the originally contemplated referral being avoided. Providers reported the service held high value for their patients and themselves. Finally, the service demonstrated cost savings when avoided medical travel expenses were considered.

Healthcare spending in Nunavut has more than tripled over the past 15 years, growing from $168.5 million in 2000 to $521.5 million in 2015 [14]. Much of this expense comes from the extensive travel many patients must undergo to attend specialist visits, a cost paid by territorial and federal governments. Consequently, eConsult exhibited even greater cost savings in Nunavut than it did in Ontario, despite having a slightly lower rate of avoided referrals (35% versus 43%). Further savings are expected when considering factors beyond the scope of this analysis. These include the out-of-pocket costs patients face in travelling great distances and missing work or school, and the potentially better health outcomes associated with quicker access to care. The precise impact of these factors is unknown and should be the subject of further study. Extrapolating from our findings, we estimate that if the eConsult service were adopted by the entire territory, with a conservative impact of 10% avoided referrals, it would demonstrate savings of over $7 million per year.

Although the use of eConsult is novel to northern communities, a few eConsult systems have been successfully implemented in remote regions. These include a tele-expertise service developed by Médecins Sans Frontières, the Knowledge Online electronic e-mail system created by the United States Armed Forces, and the Electronic Children’s Hospital of the Pacific’s store-and-forward telemedicine system. These services have demonstrated rates of satisfaction in line with the eConsult service, though their rates of referral avoidance were lower, ranging from 2% to 12% [15,16].

The chief advantage of eConsult services in Canada’s northern communities is their ability to offer electronic access to a breadth of specialties far greater than could be supported locally, given the scale and remoteness of the communities there. As such, licensing policies that restrict interjurisdictional provision of care can act as a barrier to implementation. Such was the case when telemedicine services were first introduced in the 1990s, as specialists aiming to treat patients in different provinces were required to seek separate licensing for every jurisdiction in which they provided care. The eConsult service partially circumvents this limitation, as all care is delivered by the local PCP, with the specialist playing a purely advisory role. Nevertheless, as telemedicine and eConsult services continue to expand, their ability to improve access to care for remote patients will be greatly enhanced by policies supporting more flexible interjurisdictional licensing.

Our study has limitations. The Champlain BASE™ eConsult service is established in the Champlain LHIN in Ontario. While a few Nunavut-based PCPs joined eConsult as part of a pilot project, they represent only a small fraction of the service’s users. Only nurse practitioners and family physicians are currently eligible to use eConsult as PCPs, while in many Nunavut communities nurses are the sole PCPs permanently available, many of whom are not certified as nurse practitioners. As such, the eConsult user base in Nunavut may not accurately reflect the region as a whole. Changes that would allow all nurses filling a PCP role to use the service could lead to greater benefits in remote communities and merit further exploration. We did not collect patient identifiers and do not have data on the actual number of face-to-face referrals initiated as a result of eConsult, nor whether specialists’ advice was implemented by the PCP. In our analysis, we did not consider fixed costs of the system, as the service to Nunavut was offered on top of an established system in the Champlain LHIN. If we were to include fixed administration costs, which are incurred regardless of the Nunavut eConsults, the cost weighted to the case volume would have been $172.32, which is only 1.5% of the total fixed administration costs.

## Conclusion

We provide evidence that widescale implementation of eConsult in Canada’s north would greatly improve access to specialists, improve the provider experience of care, reduce health system costs and potentially have a tremendous positive impact on quality of patient care beyond access. There is an urgency to move forward with enabling policies, provider engagement, partnerships with government and commitment to implementation.
